# A Synthetic HIV-1 Subtype C Backbone Generates Comparable PR and RT Resistance Profiles to a Subtype B Backbone in a Recombinant Virus Assay

**DOI:** 10.1371/journal.pone.0019643

**Published:** 2011-05-24

**Authors:** David Nauwelaers, Margriet Van Houtte, Bart Winters, Kim Steegen, Kurt Van Baelen, Ellen Chi, Mimi Zhou, Derek Steiner, Rachelle Bonesteel, Colin Aston, Lieven J. Stuyver

**Affiliations:** 1 Virco BVBA, Beerse, Belgium; 2 Centocor Research and Discovery, Centocor, San Diego, California, United States of America; University of Leuven, Rega Institute, Belgium

## Abstract

In order to determine phenotypic protease and reverse transcriptase inhibitor-associated resistance in HIV subtype C virus, we have synthetically constructed an HIV-1 subtype C (HIV-1-C) viral backbone for use in a recombinant virus assay. The *in silico* designed viral genome was divided into 4 fragments, which were chemically synthesized and joined together by conventional subcloning. Subsequently, gag-protease-reverse-transcriptase (GPRT) fragments from 8 HIV-1 subtype C-infected patient samples were RT-PCR-amplified and cloned into the HIV-1-C backbone (deleted for GPRT) using In-Fusion reagents. Recombinant viruses (1 to 5 per patient sample) were produced in MT4-eGFP cells where cyto-pathogenic effect (CPE), p24 and Viral Load (VL) were monitored. The resulting HIV-1-C recombinant virus stocks (RVS) were added to MT4-eGFP cells in the presence of serial dilutions of antiretroviral drugs (PI, NNRTI, NRTI) to determine the fold-change in IC50 compared to the IC50 of wild-type HIV-1 virus. Additionally, viral RNA was extracted from the HIV-1-C RVS and the amplified GPRT products were used to generate recombinant virus in a subtype B backbone. Phenotypic resistance profiles in a subtype B and subtype C backbone were compared. The following observations were made: i) functional, infectious HIV-1 subtype C viruses were generated, confirmed by VL and p24 measurements; ii) their rate of infection was slower than viruses generated in the subtype B backbone; iii) they did not produce clear CPE in MT4 cells; and iv) drug resistance profiles generated in both backbones were very similar, including re-sensitizing effects like M184V on AZT.

## Introduction

Subtype C of the Human Immunodeficiency Virus type 1 (HIV-1) is accountable for over 50% of the HIV-1 infections worldwide [1], [2], [3], [4]. Some authors suggest that the global spread of subtype C might be related to a reduced virulence compared to other subtypes [4]. However, increased tourism to, and migration from, the regions where subtype C is most common, are possibly important factors for an increasing prevalence of subtype C around the world [5], [6].

An adequate resistance-profiling tool requires an assay that correctly assesses drug resistance for all HIV variants. This can be a challenge as even quasi-species in a single individual may differ up to 10% [2]. Additionally, in order to generate correct sensitive/resistant calls, the sequence interpretation algorithm needs to be able to integrate the constantly growing knowledge of resistance-associated mutations (RAMs). This should also include different possible pathways to anti-retroviral drug resistance among the different subtypes.

The phenotypic anti-HIV drug resistance assay as described by Hertogs *et al.* [7] is based on HIV-1 subtype B (HXB2), as this was one of the first HIV-1 clones isolated from the DNA of H9 cells infected with HIV-1_IIIB_ [8]. In that assay, protease and reverse transcriptase sequences from a patient virus are recombined into the subtype B backbone, deleted for the protease and reverse transcriptase sequences, and the recombinant virus is assessed for the existence of resistance to antiretroviral drugs. We wanted to investigate whether this HXB2-based system (HIV-1 subtype B) can be used to assess resistance in protease reverse transcriptase (GPRT) sequences of non-subtype B viruses.

We constructed an HIV-1 subtype C backbone as HIV-1 subtype C is the most prevalent HIV-1 subtype worldwide and therefore an important diagnostic target. Protease-reverse transcriptase amplicons were generated from HIV-1 subtype C-infected patient samples and successfully recombined into the subtype C viral backbone. The resulting viruses allowed to test for drug susceptibility in a subtype C viral context. Subsequently, GPRT amplicons were isolated from these subtype C viruses and recombined into the subtype B backbone. The resulting recombinant virus stocks were again tested for drug susceptibility allowing a comparison of the resistance profiles measured in both HIV subtype backbones.

## Materials and Methods

### 1. Samples

Eight HIV-1 clinical plasma samples with homology to subtype C in the gag-protease-reverse transcriptase region (GPRT region) were used. A written informed consent had been obtained for the samples. All samples were anonymised before transfer and use for this study. One sample had no resistance-associated mutation (RAMs) while the remaining 7 samples had at least 8 documented RAMs (range 8–29, Table 1).

**Table 1:** Overview of the clones per sample, day of harvest (subtype C and B), VL and p24 measurements as well as the corresponding resistance-associated mutations per clone (as referenced by IAS-USA, ANRS) per sample.

			log increase			Resistance associated mutations	
Sample	Clone	DTH	VL	p24	DTH	Protease	Reverse Transcriptase
1	1	14	3.60	2.10	12	10F 15V 20R 36I 43T 46I 54L 63P 69K 71I 74P 82A 84V 90M 93L	41L 44D 67N 74V 98G 101H 118I 181C 184V 190A 210W 215Y 219R 335D
	2	18	3.10	2.30	12	10F 15V 20R 36I 43T 46I 54L 63P 69K 71I 74P 82A 84V 90M 93L	41L 44D 67N 74V 98G 101H 118I 181C 184V 190A 210W 215Y 219R 335D
	3	11	3.80	2.20	-	10F 15V 20R 36I 43T 46I 54L 63P 69K 71I 74P 82A 84V 90M 93L	41L 44D 67N 74V 98G 101H 118I 181C 184V 190A 210W 215Y 219R 335D
	4	18	3.00	2.90	12	10F 15V 20R 36I 43T 46I 54L 63P 69K 71I 74P 82A 84V 90M 93L	41L 44D 67N 74V 98G 101H 118I 181C 184V 190A 210W 215Y 219R 335D
	5	11	3.10	1.50	12	10F 15V 20R 36I 43T 46I 54L 63P 69K 71I 74P 82A 84V 90M 93L	41L 44D 67N 74V 98G 101H 118I 181C 184V 190A 210W 215Y 219R 335D
2	1	7	3.70	1.90	7	10F 13V 20R 33F 36I 46I 54V 60E 63P 69K 76V 82A 89I	41L 67N 74V 101E 118I 138A 184V 190A 210W 215Y 335D 371V
	2	5	2.50	1.50	6	10F 13V 20R 33F 36I 46I 54V 60E 63P 69K 76V 82A 89I	41L 67N 74V 101E 118I 138A 184V 190A 210W 215Y 335D 371V
	3	11	2.70	1.70	7	10F 13V 20R 33F 36I 46I 54V 60E 63P 69K 76V 82A 89I	41L 67N 74V 101E 118I 138A 184V 190A 210W 215Y 335D 371V
	4	14	3.10	1.80	7	10F 13V 20R 33F 36I 46I 54V 60E 63P 69K 76V 82A 89I	41L 67N 74V 101E 118I 138A 184V 190A 210W 215Y 335D 371V
3	1	14	3.50	1.80	12	10F 13V 15V 20T 24I 33F 36I 54V 62V 63T 69K 74A 82A 93L	41L 67N 70R 74I 101Q 184V 215Y 219Q 335D
	2	7	2.90	2.40	9	10F 13V 15V 20T 24I 33F 36I 54V 62V 63T 69K 74A 82A 93L	41L 67N 70R 74I 101Q 184V 215Y 219Q 335D
	3	18	3.20	1.80	7	10F 13V 15V 20T 24I 33F 36I 54V 62V 63T 69K 74A 82A 93L	41L 67N 70R 74I 101Q 215Y 219Q 335D
	4	14	3.10	1.40	9	10F 13V 15V 20T 24I 33F 36I 54V 62V 63T 69K 74A 82A 93L	41L 67N 70R 74I 101Q 184V 215Y 219Q 335D
4	1	11	4.00	2.50	8	15V 36I 69K 89M 93L	74V 106M 335D
	2	7	3.50	1.30	7	15V 36I 69K 89M 93L	74V 106M 335D
	3	11	2.20	1.80	8	15V 36I 69K 89M 93L	74V 106M 335D
5	1	14	2.50	1.70	7	10F 13V 15V 20H 30N 33F 36I 54V 63P 69K 74S 82A 89V 93L	118I 138A 335D
6	1	14	1.70	1.40	12	10F 15V 20V 36I 46I 50V 54V 63H 69K 71V 73S 82A 85V 89V 90M 93L	41L 67G 69D 74V 98G 103N 118I 184V 215F 219Q 335D 371V
	2	18	2.80	2.10	12	10F 15V 20V 36I 46I 50V 54V 63H 69K 71V 73S 82A 85V 89V 90M 93L	41L 67G 69D 74V 98G 103N 118I 184V 215F 219Q 335D 371V
	3	7	4.10	1.60	8	10F 15V 20V 36I 46I 50V 54V 63H 69K 71V 73S 82A 85V 89V 90M 93L	41L 67G 69D 74V 98G 103N 118I 184V 215F 219Q 335D 371V
	4	16	2.40	2.50	8	10F 15V 20V 36I 46I 50V 54V 63H 69K 71V 73S 82A 85V 89V 90M 93L	41L 67G 69D 74V 98G 103N 118I 184V 215F 219Q 335D 371V
7	1	14	3.30	2.40	9	10F 15V 20R 36I 43T 46I 54L 63P 69K 71V 74P 82A 84V 90M 93L	41L 44D 67N 74V 98G 101H 118I 181C 184V 190A 210W 215Y 219R 335D
	2	14	2.20	1.70	12	10F 15V 20R 36I 43T 46I 54L 63P 69K 71V 74P 82A 84V 90M 93L	41L 44D 67N 74V 98G 101H 118I 181C 184V 190A 210W 215Y 219R 335D
8	1	6	2.50	2.20	5	-	-
Average		12.25	3.02	1.94	9.04		
Stdev		4.07	0.61	0.42	2.38		

“DTH” Days to harvest.

### 2. Viral RNA extraction

Viral RNA extraction of plasma and virus stocks was carried out on an EasyMAG (bioMérieux, Boxtel, The Netherlands) according to the guidelines of the manufacturer, starting with 600 µl input material and eluting in 25 µl. If the samples were processed for viral load determination on the EasyQ (see below), EasyQ calibrator was added along with the magnetic silica according to the guidelines of the manufacturer.

### 3. GPRT amplification and gel analysis

A One-Step RT-PCR amplification (One-Step SuperscriptIII HiFi, Invitrogen, CA, USA) was used to generate a 2.3 kb HIV-1 fragment (containing the downstream part of GAG and about two thirds of the adjacent POL region (Protease, Reverse Transcriptase and part of Integrase)) using the “3′-RT” (5′-catgagaaatatcacagtaattggagagcaatg-3′) and “5′-OUT” (5′-gcccctaggaaaaagggctgttgg-3′) primers. RNA input was 10 µl, final volume 35 µl. Subsequently, a nested PCR was performed (final volume 50 µl, DNA input 5 µl) using the (a) “3′-In” (5′-ctaggaaaaagggctgttggaaatg-3′) and “5′-In” (5′-catctacatagaaggtttctgctcc-3′) primers generating a 1.8 kb GPRT fragment for homologous recombination with the subtype B backbone, or (b) the “5′-Infusion” (5′-aatgtggaaaggaaggacaccaaatgaaag-3′) and “3′-Infusion” (5′-ctcataaccgttcggtggacctaaggact-3′) primers generating a 1.7 kb GPRT-fragment adapted for “In-Fusion” cloning in the subtype C backbone (see below). The In-Fusion amplicon is shorter. In contrast to homologous recombination, where the overlaps are preferably as long as possible, the overlapping sequences must be exactly 15 bases long in the In-Fusion assay. Both nested amplicons encode roughly the same region of the 3′-end of GAG (from aa31 of p7 onwards) but contain the same coding sequences for protease and reverse transcriptase (400 aa). To ensure identical mutation profiles, all virus stocks were sequenced to ascertain that no other non-subtype-specific mutations might influence the resistance profile (See 2.11).

### 4. Amplicon purification

Gel extraction of the 1.7 kb GPRT-In-Fusion amplicons was performed using the QIAquick Gel Extraction Kit (Qiagen), following the manufacturer's instructions. The 1.8 kb GPRT amplicons, used for homologous recombination, were purified prior to gel analysis, using the QIAquick PCR Purification Kit (Qiagen), following the manufacturer's instructions.

### 5. Design and construction of the HIV-1 subtype C backbone for In-Fusion-cloning

#### 5.1. Initial sequence design, synthesis and construction of the HIV-1 subtype C backbone

The *in silico* design of the HIV-1 subtype C backbone was based on the subtype C sequence with accession number AB023804 (www.hiv.lanl.gov). This sequence lacked part of the 3′LTR region, which was completed by adding the matching bases as present in the 5′ LTR (5′-GTGGAAAATCTCTAGCA-3′). A B*st*EII restriction site present at position 1534 (acaGGTAACCca – coding for Thr-Gly-Asn-Pro in GAG) was changed to “acaGG**G**AACCca” conserving the translation. Also an A*cc*III restriction site (TCCGGA) at position 308 (5′ LTR) was modified to **C**CCGGA for cloning purposes (see below).

#### 5.2. Synthetic production of the HIV-1 subtype C backbone

**Figure 1:**
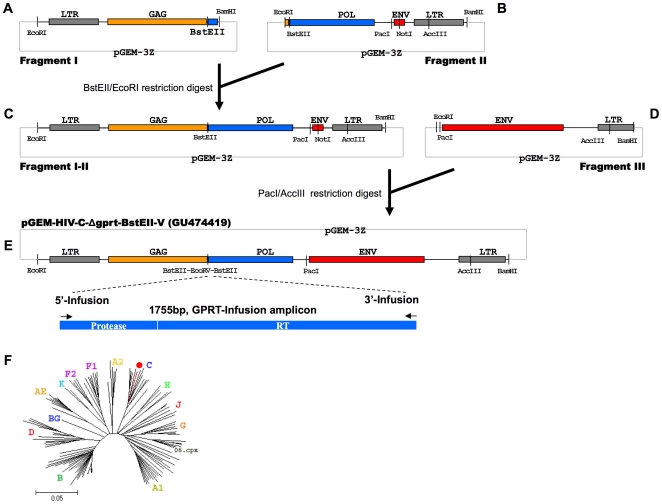
Subcloning strategy of the vector containing the HIV-1 subtype C-Δgprt backbone. Fragment I (A) and Fragment II (B) were digested using BstEII and EcoRI and religated resulting in an HIV-1 subtype C clone lacking a part of GAG, protease and reverse Transcriptase and most of ENV (Fragment I-II (C)). Fragment I-II was linearized using PacI and AccIII to insert the Env region from Fragment III (D) resulting in a final clone, pGEM-HIV-1-C-Δgprt-BstEII-V, that can be linearized using BstEII/EcoRV, ready for In-Fusion cloning with the 1.7 kb GPRT amplicon. • pGEM-HIV-1-C-Δgprt-BstEII-V+GPRT (wild type sequence).

The final design of the subtype C sequence was divided into 4 fragments (flanked by E*co*RI and B*am*HI restriction sites for cloning purposes), three of which were destined for synthesis (Fig. 1, fragments I, II, III). The synthesis of the 3 DNA fragments was performed at Centocor, CA, USA [9], [10] as follows: padded sequences were parsed into contiguous segments of equal length on both the forward and reverse strands; each segment was chemically synthesized as an oligonucleotide using GENEWRITER™ instrumentation (Centocor) and purified by reversed phase HPLC (Dionex, Sunnyvale, CA); purified oligonucleotides were assembled using proprietary gene assembly technology (Centocor, [9], [10] and cloned into a pGEM-3z vector (2743 bp) using E*co*RI and B*am*HI (Fig. 1). Vector Fragment-I (Fig. 1-A) contained an E*co*RI-B*am*HI flanking fragment of HIV-1 5′-LTR and GAG, as well as an inserted B*st*EII restriction site and a small downstream part of POL (2205 bp). Vector Fragment-II (Fig. 1-B) contained an E*co*RI-B*am*HI flanking fragment of HIV-1 GAG, as well as an inserted B*st*EII restriction site, the 3′ part of POL, a fragment of ENV (mostly deleted and replaced with a N*ot*I-containing sequence) and the 3′-LTR (3460 bp). Vector Fragment-III (Fig. 1-D) contained an E*co*RI-B*am*HI flanking fragment of the complete HIV-1 ENV and the upstream part of the 3′LTR (3412 bp). While the V3 envelope region of AB023804 was predicted to be R5-tropic according to the Geno2Pheno prediction tool (http://coreceptor.bioinf.mpi-inf.mpg.de/index.php) and Position Specific Scoring Matrices (PSSM, http://indra.mullins.microbiol.washington.edu), an R4-tropic virus was needed for the transfection assay in MT4 host cells. An envelope sequence retrieved from Los Alamos (subtype C clone C.ZA.01.01ZARP1) was predicted to be X4-tropic and was used to design Vector Fragment-III (Fig. 1-D) The fragment containing the protease and reverse transcriptase region was not synthesized but PCR-amplified from clinical samples as described above.

#### 5.3. Subcloning of the HIV-1 subtype C backbone

In a first step vector Fragment-I and vector Fragment-II were joined by subcloning the E*co*RI-B*st*EII fragment from Vector Fragment-I in Vector Fragment-II digested with the same enzymes. This resulted in an HIV-1 subtype C clone (Vector Fragment-I-II - Fig. 1-C) that had both the majority of POL (replaced by B*st*EII) and ENV (replaced by N*ot*I) deleted.

The P*ac*I-A*cc*III fragment of Vector Fragment-III (Fig. 1-D) was subcloned in Vector Fragment-I-II (Fig. 1-C) digested with the same enzymes. This resulted in a Vector Fragment-I-II-III which only had the GPRT region deleted, called “pGEM-HIV-1-C-Δgprt-BstEII”. Finally, the vector was linearized by BstEII and a small artificial sequence (5′-gtcaccgcgtgcgatatcgagcccg-3′) was inserted transforming the BstEII site into a BstEII-EcoRV-BstEII site, to reduce the background during In-Fusion and transformation into competent cells. This vector was called “pGEM-HIV-1-C-Δgprt-BstEII-V” (Fig. 1-E). The linearized vector enabled In-Fusion cloning with the 1.7 GPRT-In-Fusion patient-derived amplicons, restoring a full genome, infectious HIV-1 clone (Fig. 1-E, Genbank reference GU474419) (see below). In a phylogenetic tree, the pGEM-HIV-1-C-Δgprt-B*st*EII-V sequence (completed with a wild-type subtype C GPRT sequence) clustered together with the other HIV-1 subtype C sequences (Fig. 1-F).

### 6. Generation of full HIV-1 genomes

#### 6.1 HIV-1 subtype C backbone

The linearized pGEM-HIV-1-C-Δgprt-B*st*EII backbone was combined with the purified GPRT-In-Fusion amplicon in a molar ratio 1∶7 (final volume of 10 µl) and mixed with the dried reaction beads for In-Fusion according to the guidelines of the manufacturer (In-Fusion™ 2.0 Dry-Down PCR Cloning Kit – Clontech, Cat. No. 639607 (24 rxns), 639608 (96 rxns)), prior to transformation into bacterial cells (see 2.7.).

#### 6.2 HIV-1 subtype B backbone

In contrast to the In-Fusion strategy for the subtype C backbone, a homologous recombination event strategy was used for the subtype B backbone to generate infectious virus [7]. Here the B*st*EII-linearized pGEM-HXB2Δgprt-B*st*EII backbone was co-transfected with the 1.8 kb GPRT fragment in an MT4 cell line, resulting in a full-genome infectious virus (See below).

### 7. Transformation into MAX Efficiency® Stbl2™ cells

A total of 10 µl of diluted In-Fusion reaction mix (dilution prepared during In-Fusion cloning – see 2.6.) was added to the MAX Efficiency® Stbl2™ cells (Invitrogen, Cat. No. 10268-019) and treated according to the guidelines of the manufacturer. LB-ampicillin agar plates were incubated at 30°C for approximately 24 hours.

### 8. DNA preparation

Overnight liquid LB-ampicillin cultures (3 ml) were prepared from single colonies (*n* = 8/sample) and DNA was prepared using the PureLink HQ 96 Plasmid DNA Purification Kit (Invitrogen, Cat. No. K2100-96), according to the guidelines of the manufacturer. The plasmid integrity was checked by restriction analysis using N*de*I (New England Biolabs) and the resulting full HIV-1-C genome plasmids (*n* = 1–5 per sample, Table 1) were used for transfection.

### 9. Generation of recombinant virus stocks - Antiviral experiment

**Figure 2:**
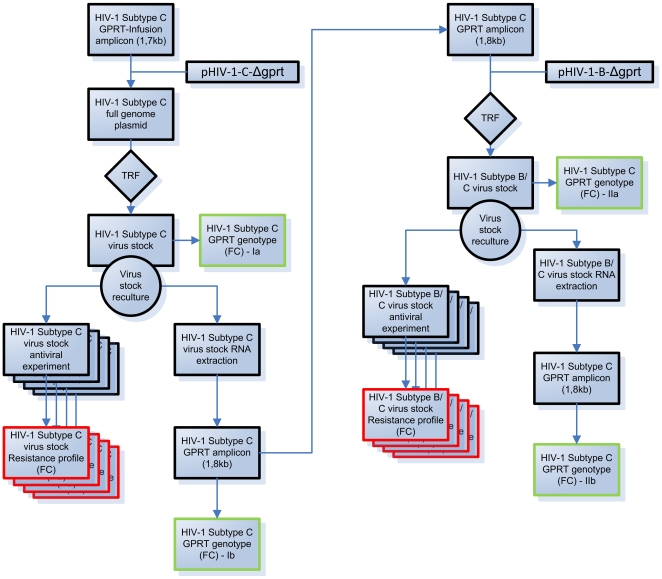
Experimental flow. Flow of the testing of the subtype C GPRT amplicons in the pGEM-HIV-1-C-Δgprt-BstEII-V (pHIV-1-C-Δgprt) and the pGEM-HXB2-Δgprt-BstEII (pHIV-1-B-Δgprt) backbones. “TRF”: transfection (Amaxa); “FC”: Fold Change; Red boxes: phenotypes; Green boxes: genotypes.

MT4 cells were transfected using the Amaxa nucleofection technology (Amaxa Biosystems, Cologne, Germany) according to the manufacturer's instruction. For the subtype C full genome plasmids: 10 µl plasmid (1 µg/µl) of the HIV-1 subtype C clones (see 2.8.) was transfected, using pulsation program A-27, into 2.5×10^6^ MT4-eGFP cells, resuspended in 100 µl solution V. Identical settings were applied for the subtype B transfection but the full genome plasmid was replaced by 1 µl pGEM-HXB2Δgprt-BstEII-linearized plasmid and 9 µl of the 1.8 kb GPRT amplicon (see 2.6.2). Transfected cells were cultured at 37°C and 5% CO2. Infection rate and CPE were monitored on a daily basis until all cells were infected (monitored by eGFP production) or until full cytopathic effect (CPE) was reached after which the recombinant virus was harvested. The resulting virus stocks were titrated and added to MT4-eGFP cells in the presence of serial dilutions of antiretroviral drugs (PI, NNRTI, N(t)RTI) to determine the fold-change in the concentration at which 50% of the virus is inhibited (IC50) compared to the IC50 of wild-type HIV-1 virus. Repeated Fold Change measurements were performed as indicated in fig. 2.

### 10. Viral load and p24 determination

Viral loads were determined on the EasyQ, using the NucliSens EasyQ HIV-1 v1.2 (Cat nr. 285 036, BioMérieux) as described by the manufacturer.

P24 measurements were performed on the MiniVidas (bioMérieux) using the Vidas P24 II (P24) kit (Cat. nr. 30117, bioMérieux) according to the guidelines of the manufacturer.

### 11 Sequencing

Sequencing reactions were performed with the Big Dye Terminator Cycle Sequencing Kit v3.1 (Cat n°. 4337457, Applied Biosystems, CA, USA). The primers used for the sequence confirmation of the pGEM-HIV-1-C-Δgprt-BstEII-V backbone after cloning are listed in Table 2-A, the primers used to sequence the GPRT amplicons are listed in Table 2-B.

**Table 2:** Sequencing primers overview.

A - pGEM-HIV-1-C-Δgprt-B*st*EII backbone sequencing primers			
Primer name	Sequence 5′-3′	Primer name	Sequence 5′-3′
HXB2-F1073	AAGACACCAAGGAAGC	HXB2-R4295	CATTGCTCTCCAATTACTGTGATATTTCTCATG
HXB2-F1494	CATAGCAGGAACTACTAGTA	HXB2-R4646	AAATTCCTGCTTGATTCCCG
HXB2-F1829	ATGACAGCATGTCAGGGAGT	HXB2-R4961	TTCCAGAGGAGCTTTGC
HXB2-F2008	GCCCCTAGGAAAAAGGGCTGTTGG	HXB2-R5504	GTTATTAATGCTGCTAGTGCC
HXB2-F2012	CTAGGAAAAAGGGCTGTTGGAAATG	HXB2-R5899	GGTACAAGCAGTTTTAGGC
HXB2-F2142	CAGACCAGAGCCAACAGCCCC	HXB2-R6147	TCTATGATTACTATGGACC
HXB2-F2261	CACTCTTTGGCAACGACCC	HXB2-R664	TTCGCTTTCAAGTCCCTGTTCG
HXB2-F2469	GGTACAGTATTAGTAGGACC	HXB2-R6834	GGACTGTAATGACTGAGG
HXB2-F2696	AATTGGGCCTGAAAATCC	HXB2-R7345	TGCGTTACAATTTCTGGGTCC
HXB2-F2871	GTACTGGATGTGGGTGATGC	HXB2-R7668	CACTTCTCCAATTGTCCC
HXB2-F3222	CCTCCATTCCTTTGGATGGG	HXB2-R8174	TTGCGATTCTTCAATTAAGG
HXB2-F324	AACTGCTGACATCGAGCTTGC	HXB2-R835	ATCGATCTAATTCTCCCC
HXB2-F3330	GTGGGAAAATTGAATTGGG	HXB2-R8477	CCGTTCACTAATCGAATGG
HXB2-F3771	GCCACCTGGATTCCTGAGTG	HXB2-R9019	GTACCTGAGGTGTGACTGGA
HXB2-F4308	AACCTGCCACCTGTAGTAGC	HXB2-R9080	CCCTTTTCTTTTAAAAAGTGGC
HXB2-F4809	TACAGTGCAGGGGAAAG	HXB2-R9100	GAATTAGCCCTTCCAGTCCC
HXB2-F5265	AGAAAGAGACTGGCATTTGG	pGEM-F10263	ATTGTTTGCAAACCTGAATAGC
HXB2-F5733	GCCATAATAAGAATTCT	pGEM-F10723	ATCTTGTTCAATCATTGATCGG
HXB2-F6013	CAGTCAGACTCATCAAGC	pGEM-F11011	CTTCCTCATCTGCAGGTTCC
HXB2-F644	GAACAGGGACTTGAAAGCG	pGEM-F11885	GTATTTCACACCGCATATGG
HXB2-F6464	CCCACAAGAAGTAGTATTGG	pGEM-F12495	TAAAGTTCTGCTATGTGGCG
HXB2-F6469	AAGAAGTAGTATTGGTAAATGTGA	pGEM-F13102	AGACAGATCGCTGAGATAGG
HXB2-F7220	CATTAGTAGAGCAAAATGG	pGEM-F13585	GGGTTGGACTCAAGACGATAG
HXB2-F761	TTTGACTAGCGGAGGCTAGAAG	pGEM-F13983	CCCTGATTCTGTGGATAACC
HXB2-F7919	GTTGCAACTCACAGTCTGG	pGEM-F14414	TATAGGGCCTCCTAGCTACC
HXB2-F8332	CTATAGTGAATAGAGTTAGG	pGEM-F14817	CTGTTTGGTGTGGCTATCAG
HXB2-F8658	GTTAGCTTGCTCAATGCCAC	pGEM-F15347	CTATTACCACTGCCAATTACC
HXB2-F8754	CCTAGAAGAATAAGACAGG	pGEM-F9857	AGTACTTGGAAGAAGCCACC
HXB2-F9000	TCCAGTCACACCTCAGGTAC	pGEM-R10289	TTGAAGCTATTCAGGTTTGC
HXB2-R1337	TCTTGTGGGGTGGCTCCTTC	pGEM-R10922	TACCCCAGTCTCAGGTTTTC
HXB2-R1682	TCTACATAGTCTCTAAAGGG	pGEM-R11228	ATACGACTCACTATAGGGCG
HXB2-R2164	GTGGGGCTGTTGGCTCTGGT	pGEM-R12019	GTCTGTAAGCGGATGCCGGGAGC
HXB2-R2414	GATAAAACCTCCAATTCC	pGEM-R12462	TATCCCCAGAGACCTTCGAG
HXB2-R2620	CATTGTTTAACTTTTGGGCC	pGEM-R12765	AAGGCGAGTTACATGATCCC
HXB2-R2817	CTTCCCAGAAGTCTTGAGTTC	pGEM-R13084	TTGCCTGACTCCCCGTCG
HXB2-R3030	GGAATATTGCTGGTGATCC	pGEM-R13534	GCAGAGCGAGGTATGTAGG
HXB2-R3273	GTACTGTCCATTTATCAGG	pGEM-R13975	CAGGAAAGAACATGTGAGC
HXB2-R3511	GGGTCATAATACACTCCATG	pGEM-R14379	TCTAGAGTCGACCTGCAGG
HXB2-R3794	CTCCCACTCAGGAATCC	pGEM-R14869	GAAAAGCAAACAAGAAAGGGG
HXB2-R3837	CTAACTGGTACCATAATTTCACTAAGGGAGG	pGEM-R15376	CAAACCACAACTAGAATGCAG
HXB2-R3879	CATCTACATAGAAAGTTTCTGCTCC	pGEM-R9795	CAAGGCCTCTCACTCTCTG

B - GPRT amplicons sequencing primers			
Primer name	Sequence 5′-3′	Primer name	Sequence 5′-3′
F1	GAGAGCTTCAGGTTTGGGG	F4	CAGACCAGAGCCAACAGCCCC
F2	AATTGGGCCTGAAAATCC	F6	GGTACAGTATTAGTAGGACC
F3	CCTCCATTCCTTTGGATGGG	F7	GTACTGGATGTGGGTGATGC
F5	CACTCTTTGGCAACGACCC	F8	GTGGGAAAATTGAATTGGG
R1	CTCCCACTCAGGAATCC	R2	GTACTGTCCATTTATCAGG
R3	CTTCCCAGAAGTCTTGAGTTC	R4	CTAACTGGTACCATAATTTCACTAAGGGAGG
R5	GGGTCATAATACACTCCATG	R7	CATTGTTTAACTTTTGGGCC
R6	GGAATATTGCTGGTGATCC	R8	GATAAAACCTCCAATTCC

The purification was performed using the DyeEX (Qiagen) purification kit, according to the guidelines of the manufacturer.

The ABI3730 XL (Applied Biosystems) performed the sequence detection and analysis was done using “Seqscape” (Applied Biosystems software).

## Results

### 1. Generation of HIV-1 subtype C recombinant virus stocks

A total of 8 different HIV-1 subtype C amplicons were processed according to the scheme depicted in Figure 2. A total of 8 colonies were picked per In-Fusion reaction (1 In-Fusion/amplicon) and sequenced, to verify a correct insert of the GPRT sequence. In total, 23 clones bearing resistance mutations, were retained for further processing (Samples 1–7, Table 1). Only one clone of sample 8 (no RAMs, Table 1) was retained and used as a subtype C wild-type reference to calculate fold-changes (see below).

### 2. Phenotypic characteristics of HIV-1 subtype C and subtype B recombinant virus stocks

**Figure 3:**
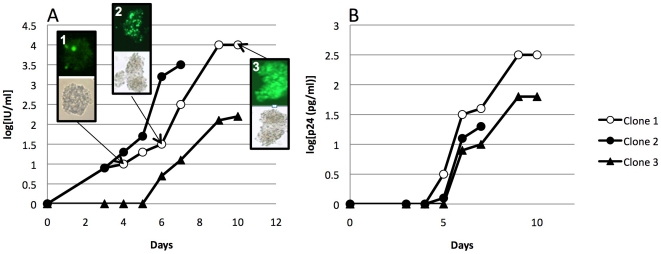
Kinetics of HIV-1 subtype C virus production. A. viral load and pictures of fluorescence (day 4(1); day 6 (2); day 11 (3)); B: p24 measurements for the three clones of sample 4.

The HIV-1 subtype C virus stock cultures were monitored on a daily basis for spread of infection, viral load (VL) and p24 production (Table 1). Some viruses replicated very fast and infected almost all cells within 7 days (*n* = 6, Table 1). A majority however, replicated slowly and needed up to 18 days to infect all cells (average time and standard deviation were 12.25 days ±4.07, Table 1). No clear cytopathogenic effect (CPE) was observed among the HIV-1 subtype C-infected cells and hence spread of infection needed to be monitored on the basis of fluorescence. As an example, the complete monitoring of the clones of sample 4 is shown in Fig. 3. Here, a gradual increase of fluorescence in cell clusters could be observed from day 4 (Fig. 3A - 1) over day 6 (Fig. 3A - 2) to the final point on day 11 where the RVS was harvested (Fig. 3A - 3). As in the example, harvesting occurred at the moment where nearly all cell clusters were completely fluorescent, which coincided with the moment at which no or a smaller increase in viral load was measured compared to the previous day. Similar curves were observed for p24 measurements. On average, a 3.02 log ±0.61 increase in VL and a 1.94 log ±0.42 increase in p24 production was observed (Table 1).

After harvesting the subtype C viruses, RNA was extracted, amplified and recombined into an HIV-1 subtype B backbone. The resulting recombinant viruses were sequenced to ensure a complete mutation analysis of the GPRT region (Fig. 2). The corresponding HIV-1 subtype B clones (Fig. 2, Table 1) grew faster (average 9.04±2.38 days until harvesting) compared to HIV-1 subtype C and gave clear CPE. One amplicon failed to generate a replicating virus after transfection in the subtype B backbone (sample 1, clone 3, Table 1).

### 3. Genotypic analysis of HIV-1 GPRT subtype C sequence in the subtype C and subtype B backbones

Identical GPRT sequences (Table 1; Fig. 2, Ia) were found for all virus stocks derived from the same amplicon except for one clone lacking 184V (Sample 3, Clone 3; Table 1). All genotypes remained unaltered during the process of re-culturing in either backbone (Fig. 2, Ib, IIa and IIb).

### 4. Antiviral drug susceptibility testing of virus stocks generated with the pGEM-HIV-1-C-Δgprt-B*st*EII vs. the pGEM-HXB2-Δgprt-B*st*EII backbones but carrying identical GPRT fragments

**Figure 4:**
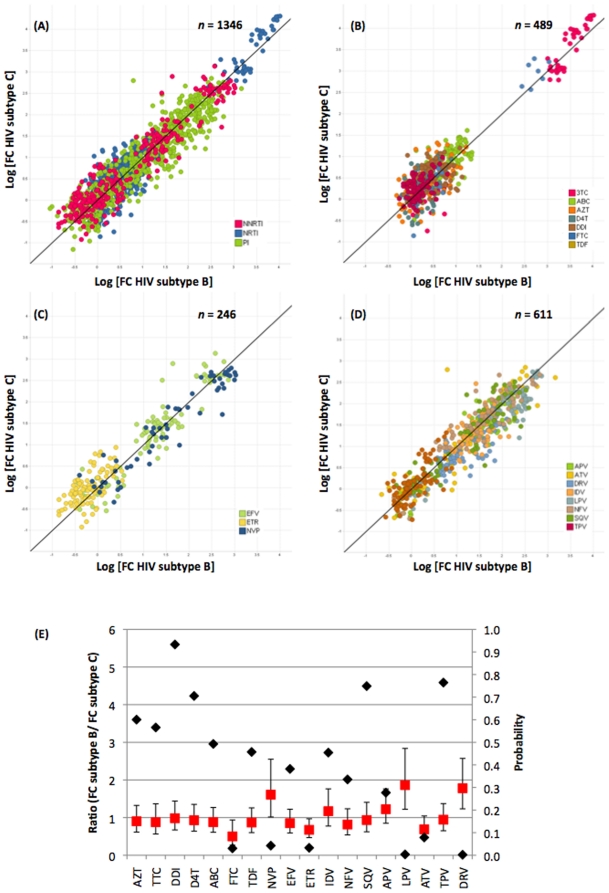
Scatter plots of FC of subtype C amplicons recombined in subtype B and subtype C backbones. (A–D) X-axis: Subtype B and Y-axis: subtype C for 1346 pairs; Black line x = y; (A) all drug classes (R^2^ = 0.88); (B) NRTIs (R^2^ = 0.88); (C) NNRTIs (R^2^ = 0.90); (D) PIs (R^2^ = 0.87); (E) Analysis of the pair-wise comparison of differences in FCs per clone and per drug, Ratio FC_Subtype B_/FC_Subtype C_ (Average, Red squares) and P-value (Black diamonds).

Fold-change (FC) values were calculated by dividing the IC50 values of the virus stocks harboring RAMs (Samples 1–7; Table 1) by the IC50 values of the corresponding backbone with wild-type amplicon (Clinical sample 8 without RAMs for the subtype C backbone, HXB2 for the subtype B backbone, Table 1). Scatter plots (1346 paired FC) showing the relationship between the FC values of the virus stocks carrying the GPRT subtype C amplicon in a subtype C backbone vs. FC values of the virus stocks carrying the GPRT subtype C amplicon in a subtype B backbone are shown in figure 4. The plots demonstrate an overall similarity in FC between virus stocks generated from a subtype C amplicons recombined in subtype B and a subtype C backbone for all drug classes (Fig. 4-A, B, C, D). Correlations were high and very similar among the three drug classes: R^2^ = 0.88 (Fig. 4-A, all drug classes), 0.88 (Fig. 4-B, NRTI), 0.90 (Fig. 4-C, NNRTI) and 0.87 (Fig. 4-D, PI). The FC of the samples analyzed covered the entire resistance spectrum from virus fully susceptible to fully resistant to one or more drugs. The ratio FC_Subtype B_/FC_Subtype C_ for most drugs was close to one (Fig. 4-E), indicating that the observed fold-change values of the GPRT amplicons in the subtype C backbone were very similar to the FC observed for that same amplicons in the subtype B backbone. However, some differences were observed. The FC ratio was significantly different from 1 (p<0.05) for emtricitabine (FTC, p = 0.031), nevirapine (NVP, p = 0.043), etravirine (ETR, p = 0.033), lopinavir (LPV, p = 0.0041) and darunavir (DRV, p = 0.002). The ratios for FTC (0.50) and ETR (0.67) suggest that, for these drugs, the FC in the subtype C backbone is higher than in the subtype B backbone, whereas for nevirapine (1.61), lopinavir (1.86) and darunavir (1.78) the opposite is observed (Fig. 4-E).

### 5. Single mutation effects on FC

**Figure 5:**
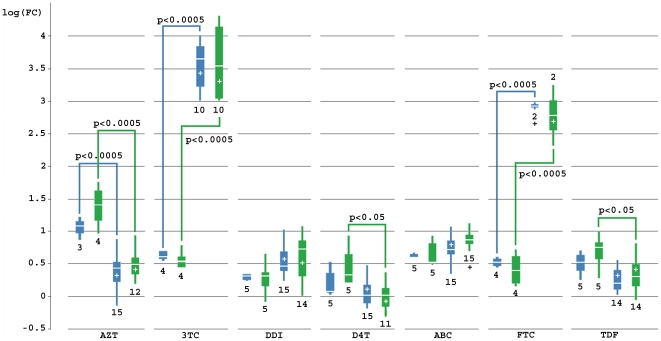
Boxplot illustrating the effect of RAM 184V on the NRTI FC in a subtype B and C backbone. Blue = HIV-1 subtype B backbone; Green = HIV-1 subtype C backbone; “+” = mutation 184V is present in RT; number under block = number of observed FC. P values have been calculated for each subtype for FC with mutation vs. FC without mutation.

Clone 3 of Sample 3 (Table 1) enabled us to investigate the effect of a single RAM (M184V in RT) on the FC of viruses with the subtype C GPRT sequence inserted in the HIV-1 Subtype B and C backbones. In RT, a change at position 184 from methionine to valine results in an increase in FC for 3TC [11], [12] and FTC [13] while it decreases the FC for AZT, d4T and TDF [14]. This effect was observed with both types of backbone as shown in Fig. 5. The increase in FC is most pronounced and highly significant (p = <0.0001) for both 3TC and FTC and for both subtype backbones. The resensitizing effect for AZT was highly significant (p<0.0001) in both subtype backbones while it was significant (p<0.05) only in the subtype C backbone for d4T (p_C_ = 0.046; p_B_ = 0.2576) and TDF (p_C_ = 0.0267; p_B_ = 0.1257). FCs for ddI and ABC were not significantly affected by the presence of 184V.

## Discussion

Synthetic biology enabled the construction of a fully replicating, infectious HIV-1 subtype C virus starting from an *in silico* design. Although promising, this approach is still in its infancy and only achieved at a relatively high cost, especially when whole functional systems are constructed [15], [16], [17], [18], [19]. This is true even for “simple/small” genomes like HIV-1. Nevertheless the expectations for Synthetic Biology go much further, evidenced by the BioBrick Foundation (www.biobricks.org), the iGEM competition (2010.igem.org) and several minimal genome projects [20], [21], [22], [23] aimed at generating a suitable chassis. We were interested in creating an HIV subtype C backbone to verify the validity of the resistance predictions performed on non-subtype B amplicons in a routine setting. As suggested by Church [24], safety and control were key operating factors and hence, the idea of creating a synthetic virus was presented to an international ethics committee that gave several recommendations. The committee concluded that identical safety measures applied to this work as to any other (research) work performed on viruses in our laboratories and that any findings needed to be reported. The committee further recommended that each research step should be carefully monitored, assessed and documented in order to (proactively) contain and resolve any possible issues.

Although the synthetic construction of the subtype C backbone might be a first for HIV, the first virus constructed in the absence of natural template was the polio virus in 2002 [25], followed in the next year by the assembly of the complete infectious genome of bacteriophage_X174 (5,386 bp) from a single pool of chemically synthesized oligonucleotides [19]. Currently larger projects are still ongoing, mostly referred to as “minimal genome” projects as discussed in Rabinow *et al.* [26] and Gibson *et al.* [23].

The aim of this study was to investigate whether the drug resistance profile of an HIV-1 subtype C GPRT amplicon is correctly assessed when introduced into an HIV-1 subtype B backbone.

To generate a unique set of fully characterized HIV-1 subtype C viruses for our study, we chose for a clonal approach by amplifying subtype C GPRT sequences from patient samples infected with HIV-1 subtype C and cloning these sequences by In-Fusion into our HIV-1 subtype C backbone. There are multiple reasons for the decision not to generate virus directly from patient samples: (a) these assays are often performed on freshly isolated donor lymphocytes [27] and we had only access to frozen plasma samples; (b) the isolation and culturing of virus from these lymphocytes is time-consuming and very labor-intensive and (c) the prolonged culture times of this kind of assay have been shown to select for subpopulations of HIV-1 variants [28] which could influence the drug susceptibility profile. Additionally, these patients have received Highly Active Anti-Retroviral Therapy (HAART) and therefore might have a rather wide range of different quasi-species, hence the clonal approach ensured a strict selection of mutations and allowed a focused resistance profiling in the subtype C background.

A recombinant virus assay strategy was used for the HIV-1 subtype B virus generation. In essence, this generated the same result as the clonal In-Fusion strategy, but is faster since the transformational step in E. coli (required to make a selection of mutations from the pool of quasi-species) could be omitted. To ensure that identical mutations were present in both backbones, the recombinant subtype B viruses were sequenced to control that no other non-subtype-specific mutations might influence the resistance profiling.

The HIV-1 subtype C backbone construct was made similar to the construct described by Hertogs *et al.* [7]. Both backbones generated X4 viruses (required for MT4 infection) and have the same regions deleted for insertion of identical GPRT amplicons. The clonal approach allowed selection of identical viral protease and reverse transcriptase sequences among clones derived from each sample. Functionality of the viral constructs (virus production) was primarily illustrated by spread of infection (monitored by CPE and fluorescence), and supported by the P24 and viral load increase. However, the nature of this cell-based assay makes a linear comparison between viral titer, viral load and P24 content impossible as investigated by Marozsan *et al.* [29]. Direct comparisons are impossible because P24 and viral load assays can only measure increase or decrease of targets, but cannot differentiate between functional and non-functional virions (hence free or incorporated RNA or P24).

Differences observed during culturing were clear (slower infection rates and nearly no CPE in subtype C viruses as compared to subtype B viruses) and are most probably due to the subtype-specific characteristics of the HIV-1 subtype C virus, which have also been observed and described by other authors [30], [31]. In fact, the observation of these subtype-related differences in our synthetic viruses, only strengthens the validity of our resistance profiling experiment as they show that our synthetic viruses behave in a similar fashion to naturally occurring HIV-1 subtype C viruses that have been studied.

The remaining question is whether HAART resistance profiles of GPRT are comparable between HIV-1 subtype B and subtype C, even if both seem to have clearly different viral kinetics e.g. regarding CPE. Directly related to this, is the obvious question whether resistance profiling of a subtype C GPRT sequence in a subtype B backbone is feasible. If differences were observed, this could have far-reaching diagnostic consequences.

This first exploratory study indicates that there were no differences in FC between phenotypic resistance assessed in the subtype B and C backbones for 13 out of the 18 drugs tested. For FTC, NVP and ETR the differences were close to the significance level of p = 0.05, whereas highly significant (p<0.05) differences were observed for LPV and DRV. While the FC in subtype C backbone seemed to be higher compared to the B backbone for FTC (ratio B/C = 0.50) and ETR (ratio B/C = 0.67), the opposite was found for NVP (ratio B/C = 1.61), LPV (ratio B/C = 1.86) and DRV (ratio B/C = 1.78). As this was a proof of concept study, the number of tested samples was limited. An analysis of a greater number of clinical HIV-1 subtype C samples is ongoing and will confirm whether the trends observed for these drugs are indeed significant.

With respect to the effect of individual mutations, FC is affected in similar ways in both subtypes as demonstrated in the analysis of the M184V mutation (increase in FC for 3TC and FTC, resensitization for AZT, d4T, TFV).

In conclusion, we successfully constructed a synthetic HIV-1 subtype C backbone for a recombinant virus phenotyping assay. The resulting recombinant subtype C viruses seemed less virulent compared to subtype B (e.g., no CPE) as has been observed in previous HIV-1 subtype C viral studies, but the generated resistance profiles were similar compared to the profiles obtained in an HIV-1 subtype B backbone for the majority of the drugs.
